# Melatonin, immune function and aging

**DOI:** 10.1186/1742-4933-2-17

**Published:** 2005-11-29

**Authors:** V Srinivasan, GJM Maestroni, DP Cardinali, AI Esquifino, SR Pandi Perumal, SC Miller

**Affiliations:** 1 Department of Physiology, School of Medical Sciences, University Sains Malaysia 16150, Kubang Kerian, Kelantan, Malaysia; 2 Center for Experimental Pathology, Cantonal Institute of Pathology, Via In Selva 24, PO Box 660, Locarno, Switzerland; 3 Departamento de Fisiología, Facultad de Medicina, Universidad de Buenos Aires, 1121 Buenos Aires, Argentina; 4 Departamento de Bioquímica y Biología Molecular, Facultad de Medicina, Universidad Complutense, 28040, Madrid, Spain; 5 Comprehensive Center for Sleep Medicine, Department of Pulmonary, Critical Care and Sleep Medicine, Mount Sinai School of Medicine, 1176 - 5th Avenue, 6th Floor, New York, NY 10029, USA; 6 Department of Anatomy and Cell Biology, Strathcona Anatomy & Dentistry Building, McGill University, Montreal, PQ, H3A 2B2, Canada

## Abstract

Aging is associated with a decline in immune function (immunosenescence), a situation known to correlate with increased incidence of cancer, infectious and degenerative diseases. Innate, cellular and humoral immunity all exhibit increased deterioration with age. A decrease in functional competence of individual natural killer (NK) cells is found with advancing age. Macrophages and granulocytes show functional decline in aging as evidenced by their diminished phagocytic activity and impairment of superoxide generation. There is also marked shift in cytokine profile as age advances, e.g., CD3+ and CD4+ cells decline in number whereas CD8+ cells increase in elderly individuals. A decline in organ specific antibodies occurs causing reduced humoral responsiveness. Circulating melatonin decreases with age and in recent years much interest has been focused on its immunomodulatory effect. Melatonin stimulates the production of progenitor cells for granulocytes-macrophages. It also stimulates the production of NK cells and CD4+ cells and inhibits CD8+ cells. The production and release of various cytokines from NK cells and T-helper lymphocytes also are enhanced by melatonin. Melatonin presumably regulates immune function by acting on the immune-opioid network, by affecting G protein-cAMP signal pathway and by regulating intracellular glutathione levels. Melatonin has the potential therapeutic value to enhance immune function in aged individuals and in patients in an immunocompromised state.

## Introduction

Aging is a complex physiological process that involves a number of biochemical reactions, with molecular changes that are manifested in single cells as well as in the whole organism. Aging reflects the sum total of all changes that occur in living organisms with the passage of time that lead to functional impairment and increased pathology. Aging is characterized by a diminished ability to respond to stress [1]. Among the many theories proposed for aging, the Oxidative Theory of Aging put forth by Harman in 1956 [2] has received wide support.

Aging is associated with a decline in immune function known as immunosenescence. This situation implies increased susceptibility to infectious diseases and cancer due to a decreased capacity of the immune system to respond to antigenic stimulation [3]. This results in altered cytokine microenvironment and impairment of both innate and adaptive immunity [4]. It is interesting to note that many hormones that are associated with maintenance of immune function also decline with advancing age and the interrelationship between the endocrine system and the immune system is considered of crucial importance in normal human physiology and in mediating age-associated degenerative diseases [5-8]. The decline in the production of a number of hormones associated with aging such as growth hormone (GH), estrogen and dehydroepiandrosterone, as well as of the pineal substance melatonin, have been proposed to play a significant role in contributing to immunosenesecence [5]. Among these, melatonin has been demonstrated to bear a general immunoenhancing effect in many animal species as well as in humans [9].

Melatonin is a natural antioxidant with significant anti-aging properties [10]. Indeed, any search for a therapeutic agent that can improve the quality of life in the elderly implies the identification of substances that have both antioxidant and immunoenhancing qualities. In this vein, the role for melatonin has been put forth [11-13] and in this paper the evidence indicating that melatonin is effective to combat age associated decline in immune function will be reviewed with the aim of advocating melatonin as a possible therapeutic agent for enhancing the quality of life in the elderly.

## Aging and immune function

Immunosenescence is associated with increased incidence of cancer and of degenerative and infectious diseases. The progressive functional T cell and B cell deficits may be the main responsible factors for age-associated disorders [4,14,15]. The involution of thymus with age results in alterations of gene expression [16]; indeed, immunosenescence is reflected at cellular, molecular and genetic levels [17]. Individuals of the same chronological age may exhibit variations in the degree of senescence associated functional impairment [18]. The role of immunity as a predictor of individual longevity in human beings has been suggested by several studies like OCTO and NONA longitudinal studies and they all reveal the existence of "immunological risk phenotype", that can predict the life span in the elderly [19].

## Aging and innate immunity

Aging affects the innate immune system [20]. In the innate immune system natural killer (NK) cells play an important role for inhibiting cancer and metastases. Longer life in centenarians has been associated with increased NK cell number, augmented interferon (IFN)-gamma production and phagocytosis [21-23]. The age-associated increases in NK cells (21) have been interpreted as a compensatory response to overcome the generally decreased immune function and has been considered helpful in arresting the growth of neoplastic cells. For example, in human NK cells from healthy subjects over 90 years of age, the ability to synthesize chemotactic cytokines upon stimulation by IL-12 or IL-2, or to express the corresponding chemokine receptors are maintained (24). However most investigators are of the opinion that functional competence of individual human NK cells declines with age [21,25]. Indeed, NK cells of aged people exhibited a diminished production of IFN gamma and chemokines in response to interleukin (IL)-2 and IL-6 [25]. Recently, Albright and her coworkers [26] found severe impairment in the production of mRNA transcripts representing several cytokines in NK/LAK cells of aged mouse. The cytotoxic capacity of NK cell is well preserved in peripheral blood of the centenarians [27].

Functional impairment of macrophages and granulocytes are reported in the elderly. Diminished intracellular phagocytic activity, degranulation and decrease in chemotactic and phagocytic activity have all been found in polymorphonuclear leukocytes of elderly individuals [24,28]. In a study in centenarians, Miyaji and his coworkers [22] found that granulocytes exhibited decreased superoxide production, irrespective of subject's health conditions. A decrease in superoxide production in elderly subjects has also been reported in other studies [29-31], the decreased production of superoxide in the granulocytes being attributed to the reduction in signal transduction in granulocytes [29]. The attenuation of Fc mediated superoxide generation and phagocytosis in the elderly has been suggested as the major factor for the age-related decline in neutrophil function [28,32]. With regard to macrophages, increased production of proinflammatory mediators like IL-1, IL-6 and IL-8 occurs in both healthy aged subjects and people showing pathological aging [33,34]. Macrophages are important for phagocytosis and destruction of microorganisms and also for cytokine production that regulates the functional ability of other cells of innate immunity.

Diminished IL-1 levels and diminished generation of reactive oxygen species (ROS) from monocytes of elderly subjects has been reported (reviewed by [35]). IL-6 (which has been termed as a "cytokine for gerontologists", [36]) increases in aged subjects [37,38]. The increase in IL-6 occurs in healthy individuals older than 85 years of age [39]. The increase in IL-6 seen in aged subjects may contribute to age-ssociated diseases [40] and mortality [41]. Plasma concentrations of soluble intercellular adhesion molecule-1 (ICAM-1) increased with age [39,42,43]. Collectively, the results suggest that it is this shift in cytokine profile that is largely responsible for triggering immunosenescence and increased morbidity and mortality in the elderly [39].

## Aging and humoral immunity

Aging results in changes in humoral immunity such as an increase in the levels of serum immunoglobulins like IgA and IgG, and decrease in the number of B and T lymphocytes [44,45]. A decline in organ specific autoantibodies together with an increase in non-organ specific autoantibodies have been found in the elderly [46]. Reduction in CD 27+ memory B cells has been reported and this correlated with low T cell number [47]. A decrease in CD5+ B cells independent of T cell decline was also reported in aging [48]. Therefore, the reduced humoral responsiveness and altered antibody-mediated defense mechanisms seen in aged individuals are explained mainly by an intrinsic primary cell deficit [49]. The ability of T cells to promote B-cell activation and antibody production may be compromised in elderly individuals, as suggested by studies using cytometric phenotypic analysis [50]. A significant decrease in IL-2 production with aging plays a role in reducing antibody production [23,49].

## Aging and cellular immunity

Aging not only causes changes in innate immunity and humoral immunity, but also causes changes in cellular immunity. A significant decrease in CD3+, CD4+, CD8+ cells and naïve T lymphocytes (CD45RA+CD4+) occurs with increase in age. An extensive review on T cell function in aging was published by Pawelec et al. [14]. With aging, alterations in signal transduction may also occur. The age-associated decline in T cell function is preceded by involution of the thymus [35]. The striking feature of T cell alterations in aging is the marked shift from naive to memory cells with an imbalance of virgin and memory cells being noted especially in CD8+ T lymphocytes [45]. Naïve T cells, which are concerned with the mounting primary immune response, are dependent upon CD28, a co-stimulatory signal for their proliferation [45]. Both the decrease in the number of naïve T cells and in their responsiveness with aging cause the decline of specific immunization response in aged individuals [51]. Large increases in CD8+ T cells with receptors for single epitopes of cytomegalovirus are common in the elderly [52]. Longitudinal studies (OCTO) suggest that the cluster of immune parameters like low CD4+ cells, an increase in CD8+ cells and a low IL-2 production are all predictive of mortality [53-55]. The decline in naïve T cells is one of the factors that cause a decreased IL-2 production[56].

## Melatonin

Melatonin (N-acetyl-5-methoxytryptamine) is formed mainly in the pineal gland of most mammals including man [57]. In the pineal gland, serotonin is converted in to melatonin through a two-step enzymatic process involving N acetylation followed by O-methylation. In humans, plasma melatonin level begins to increase steadily after 1900 h to 2300 h to attain the peak values at around 0200 – 0400 h [7]. The study of plasma melatonin among subjects of different age groups reveals a consistent decrease as aging progresses. With some exceptions [58,59] the decline of melatonin with age has been repeatedly reported [60-66]. The melatonin day/night rhythm has been found altered with phase advance in the elderly as compared to young women [67]. Great variations in the amplitude of nocturnal melatonin secretions are found among individuals suggesting that some individuals produce significantly less melatonin during lifetime than others; this may have an impact in terms of aging [7,68]. The loss of amplitude of melatonin rhythm in the advanced age is both an indication as well as a cause of age-related disturbances in the circadian pacemaker leading to chronobiological disorders [69]. This is accompanied by a general deterioration of cognitive, psychological and social functioning as well as by sleep disturbances [70-72].

The age-related impairment of the immune system first appears around 60 years of age coinciding with the decrease of plasma melatonin concentration. Indeed, melatonin has a defined immunomodulatory role both in animals and humans [13,73]. The diurnal and seasonal changes in the immune system have been shown to correlate with melatonin synthesis and secretion [74]. Melatonin is synthesized by human lymphocytes and this finding adds further support to the hypothesis that melatonin plays a role in the regulation of human the immune system [75].

## Melatonin receptors

Melatonin exerts its many physiological actions by acting on membrane and nuclear receptors although many of its actions are receptor-independent (e.g., scavenging of free radicals, interaction with cytosol proteins like calmodulin). The two melatonin receptors cloned (MT1 and MT2) are membrane receptors that have seven membrane domains and belong to the superfamily of G-protein coupled receptors [76]. Melatonin receptor activation induces a variety of responses that are mediated both by pertussis-sensitive and insensitive G proteins [77]. In the cytosol melatonin interacts with calmodulin [78]. Nuclear binding receptors have been identified in human lymphocytes and monocytes [79].

## Melatonin and immune function

In recent years much attention has been devoted to the possible interaction between melatonin and the immune system [13,73,80]. Melatonin has significant immunomodulatory roles in immunocompromised states. In 1986, Maestroni et al. first showed that inhibition of melatonin synthesis causes inhibition of cellular and humoral responses in mice [81]. Mice kept under constant light, or receiving injections of betaadrenergic blockers (propranolol) to inhibit melatonin synthesis, exhibited an inability to mount a primary antibody response to sheep red blood cells (SRBC), a decreased cellularity in thymus and spleen and a depressed autologous mixed lymphocyte reaction; all these were reversed by melatonin administration at the late afternoon [81]. Late afternoon injection of melatonin increases both the primary and secondary antibody responses to SRBC [82]. Indeed, the immunoenhancing effect of melatonin was evident only when melatonin was administered in the afternoon or in the presence of T-dependent antigenic stimulation. Since melatonin was ineffective in vitro, Maestroni and co-workers concluded that it exerts its immunostimulating effect through other neuroendocrine mechanisms in antigen-activated cells [83]. Hamsters exposed to short photoperiods had increased spleen weight and number of splenic lymphocytes and macrophages [84]. A key finding-albeit in young adult humans – with respect to the interplay of melatonin and the immune system, was the observation that the nocturnal rise of blood melatonin in humans correlated with the increase of thymic production of peptides like thymosin-1 alpha and thymulin [85].

## Melatonin and innate immunity

A number of studies support the immunoregulatory action of melatonin on the body's innate immunity [80]. Melatonin stimulates the production of progenitor cells for granulocytes and macrophages (GM-CFU) and has a general stimulatory action on hemopoiesis [86,87]. Melatonin receptors are detectable in monocyte/macrophage lineage [79] and melatonin binding to these receptors stimulates the production of GMCFU cells [88,89]. A recent pivotal study, although carried out in young adult mice, has revealed a profound, time-dependent influence of melatonin on certain cells fundamentally important to the immune system. Exogenous melatonin augments NK cells and monocytes in both the bone marrow and the spleen with a latency of 7 to 14 days [90]. As both these cells are components of the non-specific immune system, the findings suggest that melatonin could be an effective way for arresting neoplastic growth and for destroying virus infected cells. The action of melatonin on monocyte production can be partly due to its direct action on melatonin receptors or may be due to an increase of monocyte sensitivity to stimulants like IL-3, IL-4, IL-6 or GM-colony stimulating factor (GM-CSF) [88-90]. As stromal cells contain receptors for kappa opioid cytokine peptides, melatonin-induced release of opioid peptides from these stromal cells in bone marrow could be involved in the regulation of hemopoietic cell proliferation [91]. In addition to monocytes, the bone marrow precursor cells for the granulocyte lineage increase in absolute numbers after melatonin administration. The study of Currier et al. [90] revealed that melatonin increases the actual production of the GM-cell lineage and not the inter-organ trafficking of myeloid precursors. An increased activation of monocytes/macrophages by melatonin has been reported in yet another study in rodents [92]. As both macrophage cells and neutrophils form important components of the innate immune system, the stimulatory action of melatonin reflects a significant immunoenhancing property. Melatonin treatment restores the decreased total leukocyte count in peripheral blood and bone marrow of pinealectomized squirrels [93]. Macrophages have been shown to form large amounts of nitric oxide (NO) upon activation by ROS that mediate their microbiocidal properties. This excessive production of NO can be harmful to the body as it can result in the development of degenerative diseases [94]. In a recent study melatonin was found to decrease NO concentration in macrophages by suppressing inducible NO synthase expression [95]. When melatonin's effects on phagocytic activity of macrophages were tested at different concentrations, the greatest phagocytic stimulation was obtained when melatonin was added resembling the unstressed situation [96].

NK cells play an important role in immunosurveillance against neoplasia and virus infected cells [97,98]. IFN-gamma enhances NK cell activity [99]. An observation of potentially high prophylactic significance, was the demonstration that exogenous melatonin given acutely at 1800 h to young healthy males increased their responsiveness to IFN while the chronic administration of melatonin augmented the spontaneous NK cell activity and also the circulating number of NK cells [100]. The increased NK cell number brought about by melatonin administration was attributed partly to the increased production of cytokines by melatonin-stimulated T helper cells. IL-2, IL-6, IL-12 and IFN-gamma have all been suggested as the possible cytokines that mediate melatonin-induced increase of NK cell number [90]. T helper cells contain melatonin receptors that presumably mediate melatonin action in releasing cytokines [101-103].

## Melatonin and cytokine production

Melatonin has been proposed to regulate the immune system by affecting cytokine. production in immunocompetent cells [104]. Melatonin enhances the production of IL2, IFN-gamma and IL-6 by cultured human mononuclear cells [101]. Melatonin, by. activating monocytes [105], increases the production of IL-1, IL-6, TNF-alpha and ROS. Melatonin also increases IL-12 production by monocytes [105]. Repeated stimulation of T helper (Th) cells in the presence of IL-12 causes Th cells to differentiate into Th1 cells, which produce IL-2 and IFN-gamma and are particularly effective in enhancing immune responses that involve macrophages and other phagocytes. Melatonin augments IFN-gamma production by Th1 cells [104]. The enhancement of NK cell activity by melatonin is attributed to the increased production of IL-2 and IL-12 [104,106,107].

Human lymphocytes themselves play an important role in stimulating IL-2. production in an autocrine or paracrine fashion [75]. After melatonin treatment, up-regulation of gene expression for TGF-β, M-CSF, TNF-α, and stem cell factor (CSF) in peritoneal exudate cells, and the level of gene expression of IL-1β, M-CSF, TNF-α, IFN-γ, and SCF in splenocytes were reported [108]. Melatonin's immunoenhancing effect depends upon its ability to enhance the production of cytokines as well as its anti-apoptotic and antioxidant action. As a functional impairment of macrophages and granulocytes (as shown by the diminished intracellular phagocytic activity, degranulation and decrease in chemotactic activity) has been reported in the elderly [28,44] and a parallel decrease in melatonin production occurs [60-66] it may not be unreasonable to speculate that immunosenescence can be partly attributed to a decreased production of melatonin. To restore the defective phagocytic function the use of adjuvants with immunizations and nutritional supplements has been proposed [109].

Micronutrients like zinc, selenium and vitamin E play a vital role in phagocytic function [110]. Since melatonin can stimulate the immune response and correct immunodeficiencies by causing up-regulation of cytokine production it can be used therapeutically for correcting the immunodeficiency state associated with aging.

## Melatonin and cellular and humoral immunity

Besides its stimulatory action on the production of several cytokines that regulate immune function, melatonin's immunoenhancing properties have been attributed to a direct action on the immunocompetent cells (e.g. granulocyte-macrophage cells, NK cells and lymphocytes). Earlier studies demonstrated that the thymus is a primary target of melatonin's action. The thymus is an organ of youth in mammals, yet any influences on the thymus in youth will have profound effects on the immune system of elderly mammals. A milestone, earlier demonstration revealed that pinealectomized, young mice underwent accelerated involution of the thymus [111]. The presence of melatonin binding sites in membrane preparations of non-mammalian (duck) thymus has also been reported [112]. Mice kept under constant light, or administered with beta-adrenergic blockers exhibited decreased cellularity of thymus and spleen that was reversed by late afternoon administration of exogenous melatonin [81,82,113].

The severe loss of thymocytes with age is the main cause of structural thymic atrophy and thymic weight loss. Melatonin administration increased the total number of thymocytes in old mice [114]. In that study, thymic cell number in 2 months-old mice was 12.6 × 10^7^, while it dropped to 7.3 × 10^7 ^cells in 24 months-old animals; in melatonin treated old mice the total number of thymocytes was 9.1 × 10^7 ^cells [114]. This protective effect of melatonin on thymocytes was attributed to its antiapoptotic action. Melatonin inhibited glucocorticoid- or hydroxyl radical-induced thymic apoptosis [115,116]. The reversal of age-associated thymic involution by melatonin added further support to the concept that melatonin can be a potential therapeutic agent for correcting immunodeficiency state associated with aging and possibly other immunocompromised states like severe stress [117]. Finally, Yu et al. [118] have demonstrated that orally administered melatonin can substantially promote the survival (anti-apoptosis) of precursor B lymphocytes (responsible for humoral immunity) in the B lymphocyte generating site, i.e., the bone marrow. This indicates that melatonin treatment can boost the survival of mature B cells which are the functional elements in humoral immunity.

## Melatonin and T lymphocyte function

Melatonin enhances both cell-mediated and humoral immunity. The administration of melatonin to normal or immunocompromised mice elevated in vitro and in vivo antibody responses [73]. The immunoenhancing effect of melatonin involves opiod peptides; melatonin stimulates Th cells to secrete opiod peptides that have upregulatory effects on a variety of immune cells [73]. According to Nelson and Drazen [119], melatonin is a part of a complex physiological system that coordinates reproductive, immunological and other physiological processes to cope up with energetic stressors during winter. Studies in birds also indicate that melatonin stimulates both cellular and humoral responses and that the response involves opiate intermediates [120,121].

The immunostimulatory role of melatonin is exerted mainly on Th cells and on T lymphocyte precursors. There is a possibility that melatonin could act as an autocoid in bone marrow as shown by the demonstration of melatonin synthesis in bone marrow cells of mice and humans [122]. The existence of specific melatonin binding sites inlymphoid cells provides evidence for a direct effect of melatonin in the regulation of the immune system. By using the melatonin agonist 2 [^125^I]-melatonin high affinity binding sites and a signal tranduction pathway for melatonin have been characterized in human lymphocytes [123,124]. Melatonin also counteracted the inhibitory effect of prostaglandin E2 on IL-2 production in human lymphocytes via its MT1 membrane receptor [125]. Melatonin augments CD4+ lymphocytes and decreases CD8+ lymphocytes in rat submaxillary lymph nodes [126]. Collectively, these studies indicate that melatonin possesses important immunoenhancing properties and suggest that melatonin may favor a Th-1 response. During the natural history of human immunodeficiency virus type I (HIV-1) infection, an impairment of IL-12 production precedes a switch from a Th-1 to a Th-2 stage of cellular immunity. A recent study indicated a correlation of serum levels of melatonin and IL-12 in a cohort of 77 HIV-1 infected individuals, the decreased levels of serum melatonin found in HIV-1-infected individuals being possibly instrumental in the impairment of Th-1 immune response [127].

Besides the release of proinflammatory Th-1 cytokines, such as IFN-gamma and IL2 administration of melatonin to antigen-primed mice increased the production of IL10, indicating that melatonin can also activate anti-inflammatory Th-2-like immune responses in certain circumstances [128]. Therefore, it is not yet clear whether melatonin acts only on Th-1 cells or also affects Th-2 cells. This is an important subject as the Th-1/Th-2 balance is significant for the immune response [73]. Relevant to this, melatonin treatment suppressed the subsequent in vitro stimulation by the mitogenic agents LPS (that stimulates B cells) and Con A (that stimulates T cells) in submaxillary lymph nodes [126]. In addition, an inhibitory influence of melatonin on parameters of the immune function has also been demonstrated, i.e., in human NK cell activity, DNA synthesis, IFN-gamma and TNF-alpha synthesis, as well as the proliferation of T lymphocytes and lymphoblastoid cell lines were depressed by melatonin [73]. Melatonin can correct immunodeficiencies secondary to acute stress, viral diseases and drug treatment. In immunodepressed conditions, the immunoenhancing action of melatonin seems to be restricted to T lymphocyes [129]. In conditions of immunodeficiency, as in other pathologies and the normal, melatonin appears to favour a Th1 lymphocyte response [108]. Finally, a recent study (130) has estabished a significant role for melatonin, i.e., as an adjuvant with vaccination in sheep afflicted with ovine footrot, indicating that this agent clearly has significant benefits in health maintenance and disease treatment.

## Mechanism of action of melatonin in immune responses

Studies by Drazen and Nelson [102] indicated that melatonin receptor subtype MT2 but not MT1, is involved in melatonin-induced enhancement of cell-mediated and humoral function in mice. cAMP signal transduction plays an important role in regulating lymphocyte function and this pathway appeared to be abnormal in aged mice[131]. Melatonin antagonized partially forskolin-induced increase of cAMP levels of lymphocytes; indeed, G1 protein coupled adenylate cyclase-cAMP signal pathway may be one of the important mechanisms for the anti-inflammatory immunoregulation by melatonin [132]. Melatonin enhanced significantly met-enkephalin in 2 and 11 months old mice, and the effect was blocked by nifedipine, a Ca2+ antagonist [132]. This suggests that melatonin promotes the production of met-enkephalin through L-type Ca2+ channel. Melatonin-induced immunoregulation may depend upon immuno-opiod interaction [133].

It has been suggested that Th-1 responses are readily transformed into Th-2 dominance through depletion of intracellular GSH [134]. GSH in its reduced form is the single most important protective and regulatory antioxidant in cells. The work of Peterson and his coworkers [135] showed that depletion of glutathione from antigen presenting cells in vivo resulted in lowered Th-1 activity and higher Th-2 activity. Murata et al. [136,137] showed that oxidized macrophages exhibited higher levels of oxidized glutathione as they polarized to type Th-2 cells. Thus the immune activity can have Th-1 or Th-2 characteristics depending upon the relative antioxidant status of the cells.

Since melatonin stimulates the production of glutathione [138] its immunoenhancing role may be partly due to its influence on the maintenance of intracellular glutathione level. Indeed, melatonin acts as a hypnotic-chronobiotic [139,140] with cytoprotective properties [141,142] as well as an immunoenhancing agent. Indeed, melatonin not only acts as a hypnotic-chronobiotic with cytoprotective properties but also as an immunoenhancing agent. Melatonin provides a functional link between the neuroendocrine and immuno-hematopoietic systems [143].

Recent studies reveal that not only melatonin but also its oxidation product N1 acetyl-N2-formyl-5-methoxykynuramine (AFMK) is very effective in acting onneutrophils [144,145]. Both melatonin and AFMK have been shown to inhibit IL-8 release from neutrophils and AFMK has been found to be more active than melatonin in this aspect. The production of TNF-alpha by neutrophils is also inhibited by melatonin and AFMK. Since TNF-alpha and IL-8 contribute to the severity of inflammatory conditions [146], the finding of melatonin inhibiting the release of IL-8 and TNF-alpha assumes significance for it may help to reduce acute and chronic inflammation. Neutrophils are more responsive than monocytes to AFMK suggesting that melatonin biosynthesis and metabolism participate in the chemical communication among leukocytes. Melatonin may be effective in optimizing intrinsic immune responses rather than acting simply as an antioxidant [147]. Dietary supplementation of melatonin has been shown to change mRNA levels of many genes and to arrest the attenuated immune responses associated with senescence [147].

## Melatonin and season-dependent immune function

A number of recent studies point out that seasonal changes exert influence on immune function and melatonin may play an important role in this aspect. Seasonal changes of immune function in animals are mediated by the duration of melatonin secretion, which acts as a photoperiodic signal [119]. Such seasonal changes in immune function have been observed in humans also. Increased production of proinflammatory cytokines IFN-gamma and alpha occurred during winter [148]. Highest production of IL-6 was reported in healthy volunteers during autumn/winter season [149]. In humans the seasonal changes in immune functions can be mediated by the changes in duration of melatonin secretion. Seasonal changes in cytokines like IL-6, IFN-alpha, IFN or the balance of Th-1 and Th-2 response can account for seasonal changes in mood and behavior, such as Seasonal Affective Disorder.

## Summary

The age-associated decline in immune function, known as immunosenescence, is characterized by a decrease in the functional activity of NK cells, granulocytes and macrophages. There is significant reduction in IL-1 and diminished generation of ROS from monocytes. In addition, there is an increase of IL-6 production. Besides causing changes in innate immunity, aging is associated with changes in cellular and humoral immunity. Decreases of CD3 and CD4 and increases of CD8 cells occur in elderly individuals. The decrease in IL-2 production that occurs during aging causes a reduced antibody formation. Melatonin seems to play a significant immunomodulatory role. Melatonin enhances both innate and cellular immunity. It stimulates the production of progenitor cells of granulocytes and macrophages and of NK cells. Production of IL-2, IL-6 and IL-12 is stimulated by melatonin. Increased T-helper production, particularly of CD4+ cells, occurs after melatonin supplementation. Melatonin decreases CD8+ cells. Melatonin may act through the immune-opiod network. The regulation of immune function by melatonin appears to involve cAMP signal transduction, L-type Ca2+ channels and glutathione. The seasonal changes in immune function observed in animals and humans are likely to be mediated by the changes in the duration of melatonin secretion.
